# State-of-the-art CT and MR imaging and assessment of atherosclerotic carotid artery disease: standardization of scanning protocols and measurements—a consensus document by the European Society of Cardiovascular Radiology (ESCR)

**DOI:** 10.1007/s00330-022-09024-7

**Published:** 2022-10-04

**Authors:** Luca Saba, Christian Loewe, Thomas Weikert, Michelle C. Williams, Nicola Galea, Ricardo P. J. Budde, Rozemarijn Vliegenthart, Birgitta K. Velthuis, Marco Francone, Jens Bremerich, Luigi Natale, Konstantin Nikolaou, Jean-Nicolas Dacher, Charles Peebles, Federico Caobelli, Alban Redheuil, Marc Dewey, Karl-Friedrich Kreitner, Rodrigo Salgado

**Affiliations:** 1 Department of Radiology, University of Cagliari, Cagliari, Italy; 2 Division of Cardiovascular and Interventional Radiology, Department of Biomedical Imaging and Image-Guided Therapy, Medical University of Vienna, Vienna, Austria; 3 Department of Radiology, University Hospital Basel, University of Basel, Basel, Switzerland; 4 BHF Centre for Cardiovascular Science, University of Edinburgh, Chancellor’s Building, 49 Little France Crescent, Edinburgh, EH164SB UK; 5 Edinburgh Imaging Facility QMRI, University of Edinburgh, Edinburgh, UK; 6 Policlinico Umberto I, Department of Radiological, Oncological and Pathological Sciences, Sapienza University of Rome, Rome, Italy; 7 Department of Radiology & Nuclear Medicine, Erasmus MC, Rotterdam, The Netherlands; 8 Department of Radiology, University of Groningen, University Medical Center Groningen, Hanzeplein 1, 9713 GZ Groningen, The Netherlands; 9 Department of Radiology, Utrecht University Medical Center, Heidelberglaan 100, 3584 CX Utrecht, The Netherlands; 10 Department of Biomedical Sciences, Humanitas University, Via Rita Levi Montalcini 4, Pieve Emanuele, 20072 Milan, Italy; 11 IRCCS Humanitas Research Hospital, Via Manzoni 56, Rozzano, 20089 Milan, Italy; 12 Department of Radiological Sciences - Institute of Radiology, Catholic University of Rome, “A. Gemelli” University Hospital, Rome, Italy; 13 Department of Diagnostic and Interventional Radiology, University of Tuebingen, Tübingen, Germany; 14 Department of Radiology, Normandie University, UNIROUEN, INSERM U1096 - Rouen University Hospital, F 76000 Rouen, France; 15 Department of Cardiothoracic Radiology, University Hospital Southampton, Southampton, UK; 16 University Clinic of Nuclear Medicine Inselspital Bern, University of Bern, Bern, Switzerland; 17 Institute of Cardiometabolism and Nutrition (ICAN), Paris, France; 18 Department of Cardiovascular and Thoracic, Imaging and Interventional Radiology, Institute of Cardiology, APHP, Pitié-Salpêtrière University Hospital, Paris, France; 19 Laboratoire d’Imagerie Biomédicale, Sorbonne Universités, UPMC Univ Paris 06, INSERM 1146, CNRS 7371, Paris, France; 20 Department of Radiology, Charité - Universitätsmedizin Berlin, Charitéplatz 1, 10117 Berlin, Germany; 21 Department of Diagnostic and Interventional Radiology, University Medical Center, Mainz; Langenbeckstraße 1, 55131 Mainz, Germany; 22 Department of Radiology, Antwerp University Hospital & Antwerp University, Holy Heart Lier, Belgium

**Keywords:** Carotid artery diseases, Consensus, CT angiography, MR, Atherosclerotic plaque

## Abstract

**Abstract:**

The European Society of Cardiovascular Radiology (ESCR) is the European specialist society of cardiac and vascular imaging. This society’s highest priority is the continuous improvement, development, and standardization of education, training, and best medical practice, based on experience and evidence. The present intra-society consensus is based on the existing scientific evidence and on the individual experience of the members of the ESCR writing group on carotid diseases, the members of the ESCR guidelines committee, and the members of the executive committee of the ESCR. The recommendations published herein reflect the evidence-based society opinion of ESCR. We have produced a twin-papers consensus, indicated through the documents as respectively “Part I” and “Part II.” The first document (Part I) begins with a discussion of features, role, indications, and evidence for CT and MR imaging-based diagnosis of carotid artery disease for risk stratification and prediction of stroke (Section I). It then provides an extensive overview and insight into imaging-derived biomarkers and their potential use in risk stratification (Section II). Finally, detailed recommendations about optimized imaging technique and imaging strategies are summarized (Section III). The second part of this consensus paper (Part II) is focused on structured reporting of carotid imaging studies with CT/MR.

**Key Points:**

*• CT and MR imaging-based evaluation of carotid artery disease provides essential information for risk stratification and prediction of stroke.*

*• Imaging-derived biomarkers and their potential use in risk stratification are evolving; their correct interpretation and use in clinical practice must be well-understood.*

*• A correct imaging strategy and scan protocol will produce the best possible results for disease evaluation.*

## Introduction and purpose of this document

In the last 20 years, new evidence has been added to the understanding of the pathophysiology of the carotid-related stroke occurrence, by introducing the concept of the carotid artery vulnerability related to the plaque’s features. In the same period, a significant evolution of imaging techniques has occurred which not only allows for routine quantification of the degree of stenosis but also enables the assessment of the plaque composition and the detection of features of vulnerability.

The European Society of Cardiovascular Radiology (ESCR) is the European specialist society of cardiac and vascular imaging. This society’s highest priority is the continuous improvement, development, and standardization of education, training, and best medical practice, based on experience and evidence. The present intra-society consensus is based on the existing scientific evidence and on the individual experience of the members of the ESCR writing group on carotid diseases, the members of the ESCR guidelines committee, and the members of the executive committee of the ESCR. The recommendations published herein reflect the evidence-based society opinion of ESCR.

We have produced a twin-papers consensus, indicated through the documents as respectively “Part I” and “Part II.”

The first document (Part I) begins with a discussion of features, roles, indications, and evidence for CT and MR imaging-based diagnosis of carotid artery disease for risk stratification and prediction of stroke (Section I). It then provides an extensive overview and insight into imaging-derived biomarkers and their potential use in risk stratification (Section II). Finally, detailed recommendations about optimized imaging technique and imaging strategies are summarized (Section III).

The second part of this consensus paper (Part II) is focused on structured reporting of carotid imaging studies with CT/MR.

## Section I: Current concepts in imaging-based risk stratification of ischemic stroke: an introduction

### The use of stenosis severity to stratify for stroke risk

Stroke is one of the most important causes of morbidity and mortality worldwide, responsible for 5% of the global loss of disability-adjusted life years and over 10% of deaths worldwide. The current annual incidence of stroke in Europe and the USA is about 200 per 100,000 population, with 80% of strokes being ischemic [1, 2].

In 1952, C. Miller Fisher demonstrated that atherosclerotic disease of the carotid artery is one of the leading causes of ischemic stroke. Consequently, he suggested the treatment of carotid artery disease as the method of choice to prevent cerebrovascular events [3].

Before the CT/MR-era, the only available imaging technique to investigate the whole trajectory of the carotid arteries in vivo was invasive angiography. Ultrasound remains an important initial imaging modality for initial assessment (Table 1) but is reserved for evaluation of the carotid bulb region. Angiography has the inherent limitation that only luminal information, which only allowed evaluation of the degree of stenosis and presence of surface irregularities, could be obtained. Consequently, the first landmark trials performed in the 1980s were only based on the stenosis severity as chosen parameter to stratify the stroke risk, measured on 2-dimensional invasive carotid angiography. The North American Symptomatic Carotid Endarterectomy Trial (NASCET) [4] and the European Carotid Surgery Trial (ECST) [5] trials showed that revascularization in symptomatic patients with a severe degree of stenosis significantly reduced the occurrence of cerebrovascular events. As such, in the following years, the stenosis severity was incorporated into guidelines as the primary parameter to select patients for surgical therapy (together with a simplified classification of the clinical status of the patient as symptomatic or asymptomatic). According to NASCET, luminal stenosis is classified as “mild” (0–49%), “moderate” (50–69%), and “severe” (70–99%) [6, 7]. In this manuscript, we will use the same terminology when referring to amount of stenosis.

**Table 1:** Correlation between internal carotid artery stenosis and ultrasound velocity measurements. *ICA*, internal carotid artery; *PSV*, peak systolic velocity; *EDV*, end-diastolic velocity; *CCA*, common carotid artery

ICA stenosis (%)	ICA PSV (cm/s)	ICA EDV (cm/s)	PSV ratio (ICA/CCA)
Normal	< 125	< 40	< 2.0
< 50	< 125	< 40	< 2.0
50–69	125–230	40–100	2.0–4.0
> 70	> 230	> 100	> 4.0
Near-occlusion	Variable	Variable	Variable
Occlusion	-	-	Not applicable

### Looking beyond stenosis: contemporary concepts

Over the past 25 years, there has been a shift away from this previously accepted paradigm that arteries are merely conduits for blood flow, with the degree of stenosis as the sole key parameter to assess disease significance and severity.

Extensive research, including animal models of atherosclerosis and observations using modern imaging techniques such as ultrasound, computed tomography angiography (CTA), and magnetic resonance (MR) without or with angiography (MRA), revealed that vessel walls actually integrate complex dynamic structures which are involved in the regulation of blood flow. The previous—simplified!—understanding of atherosclerosis, being a process leading to a progressive decrease of lumen caliber resulting in blood flow-limiting lesions, was now challenged by the identification of multiple complex molecular and cellular processes driven by the endothelium that influence the condition of the remaining vessel wall elements. Early atherosclerotic processes result in a relatively predictable asymptomatic progression of disease, followed later by unpredictable manifestations that possibly result in downstream ischemic sequelae [8].

In particular, a deeper understanding of the role of atherosclerotic carotid plaques, their biochemical and ultrastructural composition, and their geometry emerged. Several studies have shown the importance of plaque characterization in both symptomatic and asymptomatic patients [9, 10]. Consequently, the assessment of stroke risk using only the degree of stenosis, as in the previously mentioned NASCET and ESCT clinical trials, seems outdated now as the acquired knowledge gained over the last years about additional imaging biomarkers is not recognized or incorporated. Moreover, the potential impact of the stenosis degree has been further questioned by the understanding of the positive remodeling phenomenon (further detailed in Part II of this document), in which a substantial atherosclerotic plaque can develop without any or very limited repercussion on luminal stenosis. In other organ systems including the coronary arteries, the critical impact of plaque features other than stenosis has been extensively validated [11–15]. Based on this experience and increasing knowledge, the incorporation of additional imaging parameters reflecting multiparametric plaque features into the assessment of the carotid arteries allows for a more accurate risk estimation for ischemic stroke and improves individual patient management.

### Imaging work-up: for whom?

#### ESCR Consensus Statement I

*In asymptomatic patients, CT or MR should be the second-line imaging techniques that is always performed after an initial ultrasound exam has identified a severe stenosis, plaque features possibly correlated to increased stroke risk or was not conclusive.*

*In symptomatic patients, CT or MR can be considered the first-line imaging technique.*

In the definition of the imaging work-up, a key point is the definition of the status of the patient as symptomatic or asymptomatic.

In *asymptomatic* patients without risk factors for carotid disease, most guidelines and health authorities do not recommend imaging for screening purposes [16, 17]. The reason is that complications due to unnecessary interventions resulting from overdiagnosis outweigh the benefit of early detected disease on a population level, partly due to the low prevalence of high-grade stenoses. Some guidelines recommend “targeted screening” in asymptomatic patients with a high-risk profile (previous acute myocardial infarction, pathological blood exams) for carotid disease due to the proven reduction of risk for stroke with revascularization in subjects with asymptomatic severe (more than 70%) carotid stenosis [18] (https://acsearch.acr.org/docs/69478/Narrative). However, there is currently no agreement on this point and discussions are ongoing.

Risk scores to enable targeted screening of cases in populations with an elevated risk of asymptomatic carotid stenosis have been developed [19] and risk prediction models can be used to select particular individuals for targeted screening to detect asymptomatic carotid stenosis, allowing improved cardiovascular risk management to prevent complications. In a recently published multi-national survey in asymptomatic patients [20], the first exam used to evaluate carotid bifurcation was ultrasound in 88.8% of cases, CT in 7%, and MR in 4.2%. If a severe degree of stenosis was found in the US analysis, a second level exam was performed (CT in 88% of cases and MR in 12% of cases).

In the case of *symptomatic* patients, work-up strategies are oriented to the identification of the causes of stroke and therefore imaging of the carotid artery is a mandatory examination. There are significant differences in the imaging strategies according to multiple parameters (geography, level of the hospital (first, second or tertiary level) and the academic / nonacademic institutions) as recently published [20]: In the case of symptomatic patients, half of the centers perform US as the first exam to evaluate carotid arteries (50.4%) followed by CT (41.6%), and then MR (8%). In symptomatic patients that are first studied with US, if a severe degree of stenosis is found, a second level exam is performed (CT in 85% of cases and MR in 15% of cases). Symptomatic persons that are admitted to a hospital with acute stroke symptoms usually undergo CT stroke imaging including a CT angiography of the head and cervical vessels with the ongoing current practice of performing frequently CT of the intracranial vessel at the admission in the symptomatic stroke [21] that could be easily extended from the intracranial level to the cervical level. These data suggest that patients with cerebrovascular symptoms could be directly assessed with second-level imaging. In this scenario, it is also important to underline that in several centers the request for CT or MR after ultrasound is oriented by the detection of a severe degree of stenosis (assessed morphologically or with the PSV values). However, recently published papers have demonstrated that carotid plaque rupture plays a key role also in patients with mild or absence of degree of stenosis (cryptogenic stroke) [10, 22]. This information suggests that also subjects that are considered with the absence of severe carotid stenosis could have a vulnerable, eccentric plaque that is the cause of the cerebrovascular symptoms [23–25].

## Section II: CT- and MR-derived plaque imaging features

### Quantifying the degree of stenosis

#### ESCR Consensus Statement II

*Historically, carotid stenosis remains primarily quantified and reported as a percentage-based luminal minimal diameter stenosis against a chosen reference diameter. The stenosis severity remains the only available validated parameter in current guidelines for treatment decision-making based on the 70% stenosis cut-off. We recommend using the NASCET method to assess carotid artery stenosis, and to clearly mention this choice in the radiology report.*

*To exploit the potential offered by modern CT and MR imaging and post-processing software, cautious inclusion of other additional approaches is possible, available, in order to incorporate all morphological information for better risk-stratification of the individual patient. Nevertheless, no guidelines-supported cut-off values exist for these techniques, and they must be carefully interpreted on a case-by-case basis considering all clinical and imaging information.*

As previously explained, the NASCET and ESCT trials both demonstrated the ability of carotid endarterectomy (CEA) to prevent strokes and death in symptomatic patients with severe carotid stenosis. Specifically, these trials used the percentage degree of stenosis in the internal carotid artery (ICA) as the lead parameter to guide a relatively simple and reproducible strategy to assess patients’ stroke risk and stratify between surgical and non-surgical therapeutical options.

**Fig. 1:**
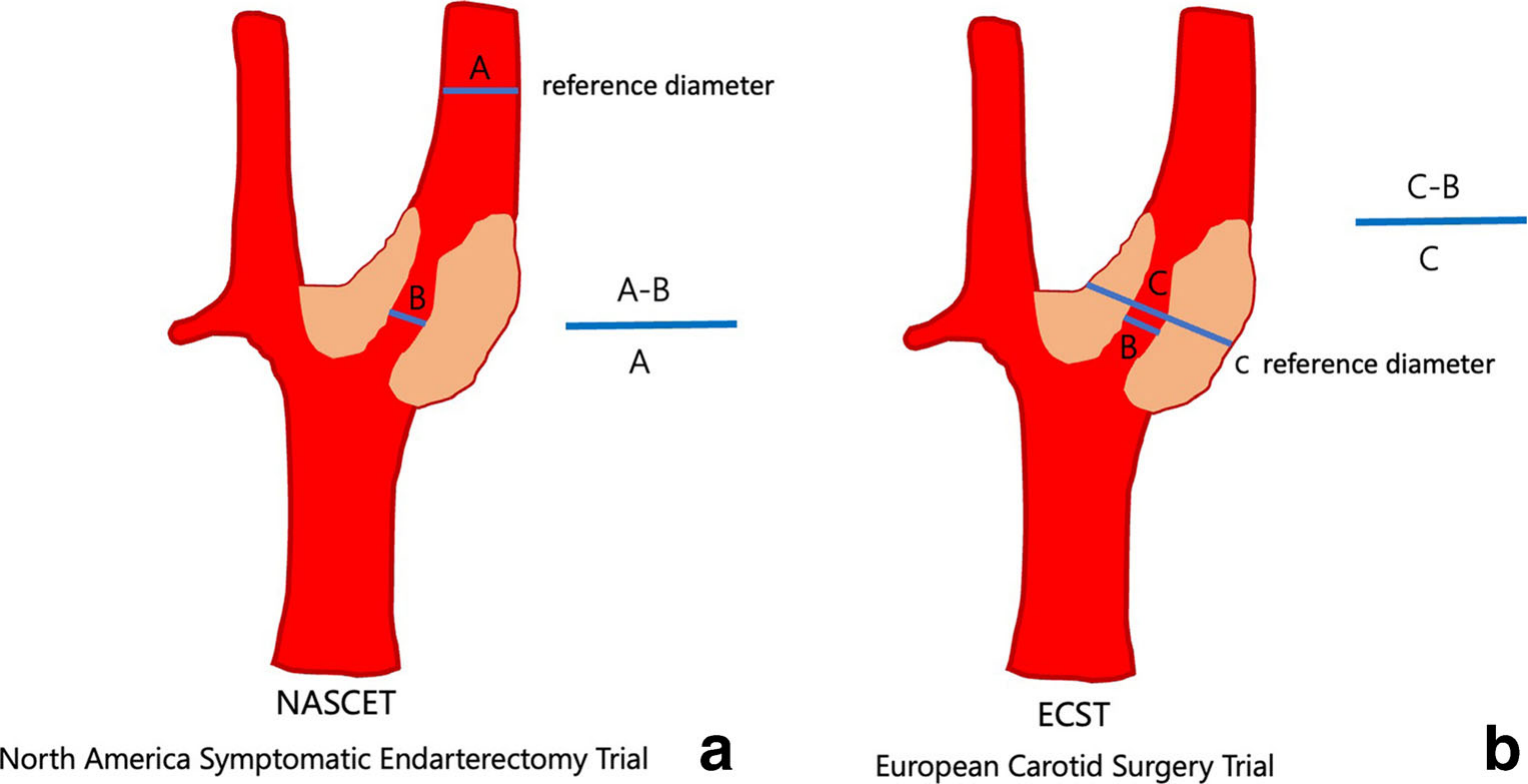
Measurement of carotid stenosis using the NASCET (**a**) and ECST (**b**) methods, revealing the difference in choice of reference diameters. While both methods use the narrowest luminal diameter at the location of stenosis, the reference diameter is in the NASCET method located well beyond the carotid bulb where the walls are parallel, while ESCT uses the outside diameter of the carotid bulb. All measurements are cross-sectional perpendicular to the long-axis of the artery, and expressed in percentage stenosis. As such, it is easy to understand that the same lesion leads to different degrees of stenosis between NASCET and ESCT. NASCET, North-America Symptomatic Carotid Endarterectomy Trial; ESCT, European Carotid Surgery Trial

In practice, the currently most used approach to quantify the degree of stenosis is the NASCET technique [26]. The NASCET and ESCT method of measuring stenosis are detailed in Fig. 1 and differ significantly. Comparing luminal stenosis at the bulbus level to the estimated anatomical bulb diameter (ESCT) yields completely different values than comparing the bulbar luminal diameter to a reference vessel diameter well beyond the region of the bulb (NASCET). Therefore, it is fundamental to indicate in every report *which quantification technique was used* (NASCET or ECST). Based on the results of a systematic review by Abbott et al, the NASCET system is the most commonly used method in guidelines [26].

**Fig. 2:**
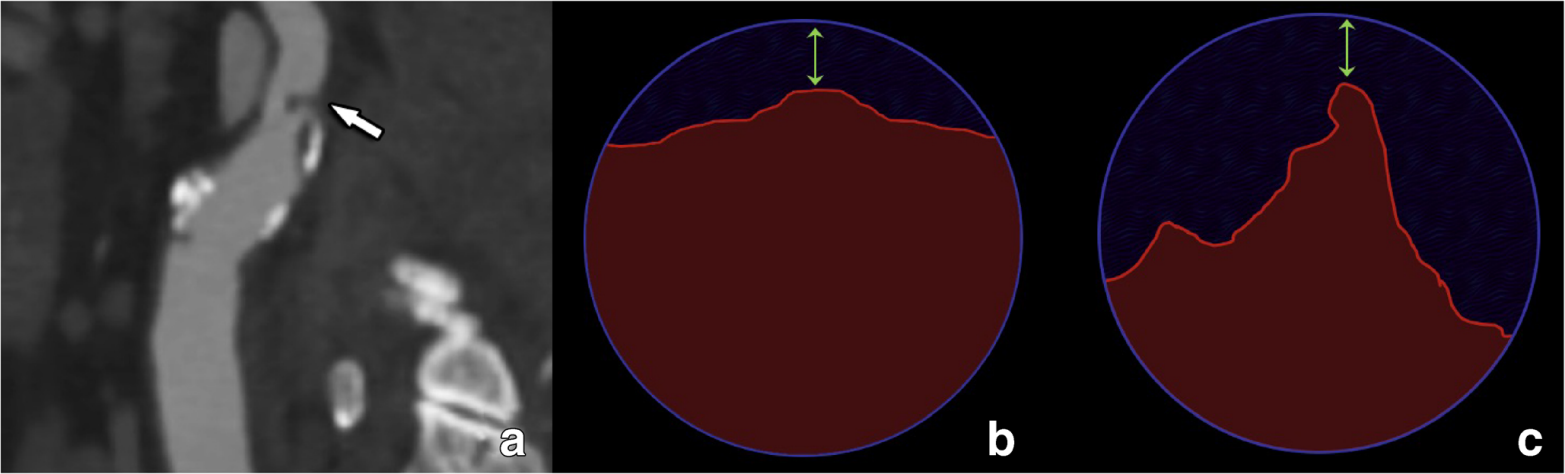
Limitations of diameter-based vs area-based stenosis assessment. A small intraluminally protruding non-calcified plaque is seen (arrow in **a**). However, as shown in panels **b** and **c**, the same luminal degree of diameter stenosis can be found in two totally discrepant situations, where different plaque morphologies produce the same narrowest luminal diameter but very different degrees of area involvement. In these instances, further description of plaque morphology and surface delineation is critical to provide a complete assessment beyond merely reporting the degree of luminal stenosis

Over the years, different approaches to stenosis quantification have additionally been suggested addressing the limitation of measurement based on projections from invasive digital subtraction angiography (DSA) as used in both the NASCT and ECST trials. These approaches include area-based stenosis or providing absolute minimal diameter values [27, 28]. These new metrics offer a different perspective of stenoses caused by plaques, taking into account all available 3D anatomical information (e.g., area-stenosis, Fig. 2). However, no guidelines-supported cut-off values exist for these techniques to guide clinical management, and they are not considered standard practice outside research settings. Furthermore, they are often cumbersome to implement in clinical practice, although recent software advances have the potential to make these measurements a more straightforward task.

### Carotid plaque components

#### ESCR Consensus Statement III

*Imaging-derived biomarkers can be obtained by means of CT and MR to provide detailed information about plaque vulnerability and to predict further cerebrovascular events. While previous guidelines have not formally adopted these new biomarkers, the current evolution points to a future multifactorial approach for the risk stratification of carotid plaques beyond the sole quantification of stenosis, incorporating all validated new biomarkers.*

Based on already existing knowledge and evidence and facing the potential of modern imaging techniques, the standardized quantification of the degree of stenosis should be supplemented with information about the underlying plaque and its characteristics as well as about vessel and luminal surface morphology to improve stroke risk stratification and treatment decision making. Consequently, a state-of-the-art imaging study of the carotid arteries should provide detailed information on the two main morphological features: plaque morphology and vessel morphology.

While various plaque components will now be discussed, they are not all a mandatory part of a routine radiological report. A practical overview of different plaque components, their clinical setting (routine vs research), and preferred imaging modality is given at the end of this paper.

#### Plaque composition: standard classification

In the following sections, a detailed overview of plaque components and characteristics within their clinical context is provided.

In 1995, the American Heart Association (AHA) published a detailed classification scheme designed to be used as a histological template for images of plaques obtained by a variety of invasive and noninvasive techniques in the clinical setting [29]. In this AHA scheme (Table 2), revised in 2000 [30], the lesions are designated by Roman numerals, indicating the usual sequence of lesion progression: evolving from an initial type I lesion to eventually type VIII with predominant fibrous tissue changes within the plaque. This classification was based on MR and CT imaging studies [31, 32]. Building further on the AHA scheme, Virmani and colleagues focused on erosion, rupture, and thinning of the fibrous cap [33]. This has become the most widely accepted system in use today [34].

**Table 2:** AHA classification and AHA-MR based classification from reference 31

	AHA classification	Carotid MR-based AHA classification from Cai et al
Type I	Initial lesion with foam cells	Near-normal wall thickness, no calcification
Type II	Fatty streak with multiple foam cell layers	
Type III	Pre-atheroma with extracellular lipid pools	Diffuse intimal thickening or small eccentric plaque with no calcification
Type IV	Atheroma with a confluent extracellular lipid core	Plaque with a lipid or necrotic core surrounded by fibrous tissue with possible calcification
Type V	Fibroatheroma	
Type VI	Complex plaque with possible surface defect, hemorrhage, or thrombus	Complex plaque with possible surface defect, hemorrhage, or thrombus
Type VII	Calcified plaque	Calcified plaque
Type VIII	Fibrotic plaque without lipid core	Fibrotic plaque without lipid core and with possible small calcifications

MR is the method of choice over CT for advanced carotid plaque analysis due to improved contrast resolution and superior tissue component analysis. However, as discussed in the next section, promising advances in CT building on good spatial resolution, the availability of advanced software tools and new techniques (e.g., spectral imaging) to extract additional information, together with improved automation and standardization, have the potential to increase the value of CT in this field. On CT, we recommend a simplified plaque classification based on attenuation density (Hounsfield units, HU) with 3 groups: low-attenuated plaques (< 60 HU), mixed plaques (60 to 130 HU), and calcified plaques (> 130 HU) [35].

#### Fibrous cap

The fibrous cap (FC) is a layer of fibrous connective tissue on the intimal surface of an atherosclerotic plaque, which is thicker and less cellular than the normal intima [36]. FC alterations are considered to be a feature of plaque vulnerability with FC thinning present in 95% of symptomatic plaques but also in 48% of asymptomatic plaques (*p* = 0.003) [37]. The importance of this parameter, first described with MR in 2000 [38], is confirmed by recent MR [39] and histopathological studies [40]. Prospective studies demonstrated that, among other plaque features, a thinning/rupture of the FC as detected with MR is associated with an increased risk for TIA/stroke [41, 42]. MR is to date likely the sole imaging technique to assess this feature non-invasively, with the most accurate characterization by CE-MR [43].

#### Plaque composition: calcium

Detection and visualization of carotid artery calcium are possible with both CT and MR. However, because of the high X-ray attenuation of calcium [44], CT is considered the reference standard for quantification and characterization of carotid artery calcification [45, 46]. Some studies have shown that carotid plaque calcification is a protective plaque feature associated with biomechanical plaque stability [47]. By extension, densely calcified atherosclerotic plaques have been described as less prone to disruption and are less likely associated with symptoms than non-calcified carotid plaques of similar size [47–50]. In a meta-analysis conducted by Kwee in 2010, clinically symptomatic plaques were found to have a lower degree of calcification than asymptomatic plaques [48]. A recent systematic review and meta-analysis by Baradaran et al confirmed that there is a negative relationship between the amount of carotid artery calcification and ipsilateral ischemia (odds ratio [OR] 0.5; 95% confidence interval [CI] 0.4 to 0.7) [51].

**Fig. 3:**
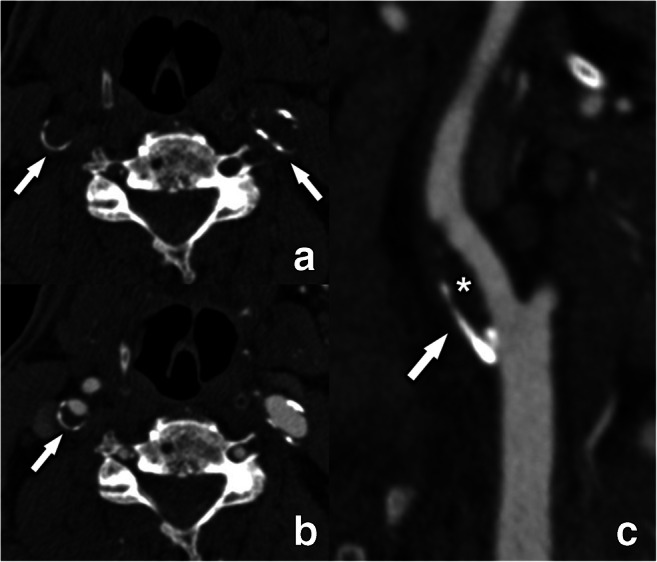
The rim sign illustrated on CT. Fine peripheral semicircular calcifications can be seen in both internal carotid arteries (arrows) on an unenhanced CT-scan at the level of the carotid bulb (**a**). After intravenous contrast (**b** and **c**), the plaque composition becomes more defined. The right internal carotid artery plaque is composed of a fine (< 2 mm thickness) semicircular adventitial calcification (arrow) and a more pronounced (> = 2mm thick) non-calcified component (asterisk), meeting the criteria for a so-called “rim sign.” The left internal carotid artery has small peripheral calcification of less than 2 mm thickness, but the non-calcified component is not large enough to qualify this plaque as a rim sign

However, other authors have suggested that the presence of calcium within carotid plaques could represent an independent marker for luminal stenosis and ischemic symptoms [52]. Recent findings indicated that calcified atherosclerotic burden is a marker of plaque instability [53, 54]. These discrepancies can be explained by the fact that vessel wall calcification may occur at different stages and pathways of atherogenesis [55–57]. Recently, it has been reported that not only the amount but also the type and chemical composition of calcium in atheromatous plaques can affect plaque stability [58]. In particular, a study conducted in 96 patients found that the “rim sign” (Fig. 3), defined as the presence of an adventitial calcification (< 2 mm thick) with internal non-calcified plaque (≥ 2 mm thickness), had a statistically significant association with intra-plaque hemorrhage (IPH); prevalence ratio = 11.9, *p* < 0.001) [59]. In this scenario, adventitial calcifications with a positive rim sign may represent a marker of high risk.

#### Plaque composition: intraplaque hemorrhage

**Fig. 4:**
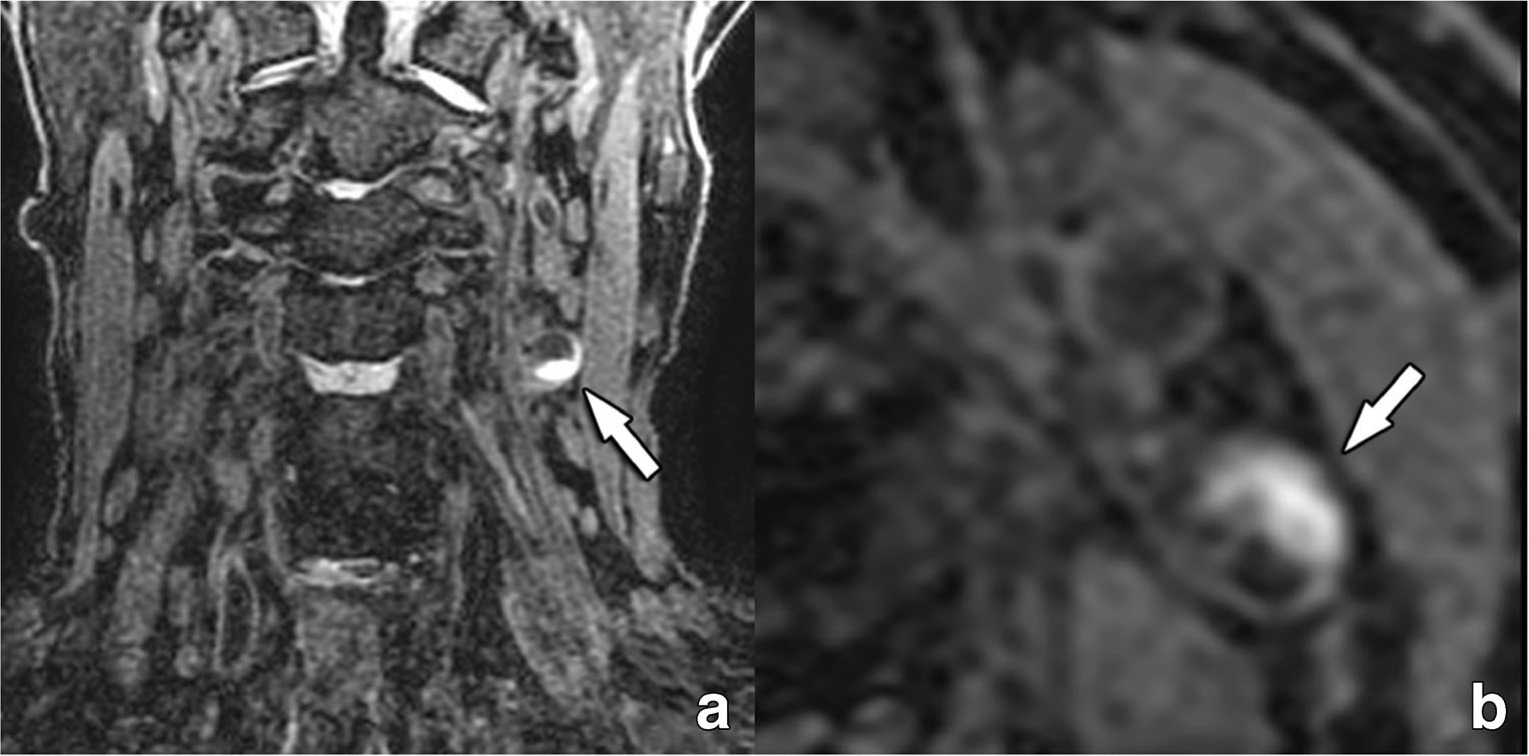
A 83-year-old female with a left-sided stroke and intraplaque hamorrhage as detected with MR. A 3D MPRAGE MR image reveals a high-intensity semi-circumferential plaque in the left internal carotid artery (arrow in **a**, **b**), corresponding to intraplaque hemorrhage. The excellent tissue contrast resolution of MR makes it the non-invasive imaging method of choice for the detection of blood degradation products in hemorrhagic plaques. MPRAGE: magnetization-prepared 180° radio-frequency pulses and rapid gradient-echo

Thirty years ago, the presence of intraplaque hemorrhage (IPH) was first described in histopathological studies performed in carotid plaques of symptomatic patients [60–62], suggesting that this feature could be considered a marker of plaque instability. With the evolution of imaging techniques, especially MR, the non-invasive detection of blood components (with e.g. high signal on T1-weighted images) within a plaque became possible. In 2003, Moody et al published a landmark paper showing that it was possible to detect IPH within carotid plaques using MR [63]. Currently, MR-detected IPH is considered the lead imaging biomarker of carotid artery plaque vulnerability (Fig. 4) [64, 65]. It is important to underline that IPH is also more prevalent in carotid arteries ipsilateral to embolic strokes of “undetermined source” [10, 22].

The prevalence of IPH in the carotid arteries in patients without severe stenosis could be used to reclassify the standard scheme of causative stroke, including plaques without severe luminal stenosis as a potential cause of stroke [66]. In a cohort study of 1190 asymptomatic subjects [67], IPH detected with MR was shown to be a high-risk factor for a subsequent cerebrovascular ischemic event, with a significantly lower event-free survival rate of patients with IPH *p* < 0.001). In a recent individual patient data meta-analysis, including 560 patients with symptomatic and 136 patients with asymptomatic carotid stenosis, the presence of IPH at baseline increased the risk of ipsilateral stroke in both symptomatic (hazard ratio [HR] 10.2; 95% CI 4.6 to 22.5) and asymptomatic (HR 7.9; 95% CI 1.3 to 47.6) patients. The authors suggested that IPH could be also detected with CT [68, 69] by considering the portion of the plaque with an attenuation value of < 25 to 30 HU. However, there is a limited specificity with CT due to the overlap in attenuation values between IPH, fibrotic, and lipid components.

#### Plaque composition: lipid-rich necrotic core

The lipid-rich necrotic core (LRNC) is an important marker of potential carotid artery plaque vulnerability. The Multi-Ethnic Study of Atherosclerosis (MESA) showed that LRNC is a predictor of cardiovascular events and offers better performance compared to traditional risk factors [70]. Another study of 214 subjects with established cardiovascular disease that were followed up for 35 months showed that the amount of lipids in a carotid artery plaque is significantly associated with an increased global cardiovascular risk (*p* = 0.002) [71]. Other studies found similar results [72, 73]. In 2013, a meta-analysis including nine studies with a total of 779 subjects found that carotid plaques with a LRNC were associated with an increased risk of cerebrovascular events (HR 3, 95% CI, 1.51–5.95) [41].

MR is the method of choice for the identification of the LRNC because of its excellent contrast resolution and feasibility to distinguish the lipid-tissue signal characteristics [74, 75] (Table 3). Some authors have also used CT to identify the LRNC. De Weert et al suggest using a threshold of < 60 HU to identify the LRNC [35]. However, it is important to realize that CT cannot itself confirm the presence of LRNC but can merely identify low-density areas that could represent LRNC.

**Table 3:** The signal of the MRI plaque elements

	T1 pre	T1 post	T2	PD	TOF
LRNC	Iso/high	Low	Iso/high	Low	Low
Fibrous cap	Iso	Iso	Mixed	Mixed	Low
Fibrous tissue	Iso/high	Very high	Iso/high	Iso/high	Low
IPH	Very high		Variable	Variable	Variable
Calcification	Low	Low	Low	Low	Low

#### Maximum wall thickness and plaque volume

Maximum wall thickness (MWT) identifies the maximum thickness of the plaque; this parameter is easy to assess and is a surrogate parameter of the plaque burden. In particular, it has been demonstrated that MWT is a better predictor of cerebral ischemic events (AUC = 0.93) compared to the classic degree of stenosis parameter (AUC = 0.81) [76].

Recently, it has become easier to quantify the volume of carotid artery plaques and plaque sub-components, largely due to the evolution of quantitative image post-processing software techniques as well as AI technology (as described even further under the “Vessel- and luminal surface morphology” section). From an algorithmic point of view, this is an easy step after plaque segmentation, as the volume of plaque components can be calculated according to attenuation density on CT or signal thresholds on MR [77–79]. It has been shown that the volume of plaque is associated with features of vulnerability [80] and that the annual progression of plaque volume is associated with the occurrence of future cerebrovascular events [79].

#### Plaque neovascularization

The importance of carotid plaque neovascularization was demonstrated in a histopathological study published in 2004. Carotid specimens of 49 patients (22 with symptomatic atherosclerosis and 27 patients with no history of cardiovascular events) were analyzed. Subjects with symptomatic carotid plaque had a denser network of vasa vasorum than patients with asymptomatic disease (33 ± 2 versus 25 ± 2 adventitial micro-vessels per 1 mm^2^; *p* = 0.008) [81]. In another longitudinal study, there was an inverse relationship between the micro-vessel density in atherosclerotic lesions and the timing of ischemic neurological events (OR 4.63, 95% CI 2.95 to 7.28, *p* < 0.001) [82].

Neovascularization can be analyzed non-invasively by advanced CT [83] and MR [84] techniques. Currently, it is not considered part of the standard clinical exam. Dynamic contrast-enhanced (DCE) MR perfusion has been introduced as a promising technique for the quantification of plaque vascularity [85] providing reproducible physiological measurements of the vasa-vasorum [86, 87]. Furthermore, plaque neovascularization can be quantified by measuring plaque enhancement on post-contrast CT images compared to pre-contrast CT. Carotid plaque enhancement (CPE) on CT is associated with the presence of neovascularization and micro-vessel density [83].

#### Plaque and peri-carotid fat inflammation

Plaque inflammation is an important parameter related to plaque atherogenesis and vulnerability involving multiple inflammatory cells. Multiple pre-clinical and cross-sectional pathological studies have demonstrated the relationship between inflammation and cerebrovascular events. Most imaging studies performed to define the presence and amount of inflammation within the plaque use nuclear medicine imaging, particularly positron emission tomography (PET). PET/CT with ^18^F-fluorodeoxyglucose ([^18^F]FDG), an analogue of glucose, can identify large vessel inflammation as seen on histology [88]. Moreover, [^18^F]FDG PET and [^18^F]FDG PET-CT can distinguish between recent culprits and asymptomatic carotid plaques in patients with atherosclerosis [89]. As demonstrated in the multi-center randomized BIOVASC trial, ^18^F-FDG PET-CT uptake can predict recurrent stroke (AUC 0.80, 95% CI 0.64 to 0.96) [90]. ^18^F-sodium fluoride, a marker of calcium metabolism and necrosis, is also able to identify culprit carotid plaques and plaques with high-risk features [91]. Beyond glucose metabolism, novel radiotracers targeting pathways involving leukocyte recruitment to sites of inflammation may offer increased specificity and concurrently provide additional insight into cardiovascular pathology [92]. For example, preliminary trials report promising results focusing on radio-compounds targeting amino acid metabolisms such as ^11^C-Methionine [93], chemokine receptors (e.g., CXCR4) with ^68^Ga-Pentixafor [94], somatostatin receptors like ^68^Ga-DOTATATE [95], mitochondrial translocator protein [96], and integrin activity like ^18^F-Galacto-RGD [97]. The envisioned wider clinical use of such PET tracers is expected to provide important mechanistic information on the composition and biological activity of these plaques.

Of note, combining these tracers with MR, in PET/MR imaging, may provide further information on carotid plaque vulnerability [98]. MR is also able to track the presence of inflammatory processes in carotid plaques through the use of ultrasmall superparamagnetic iron oxide contrast (USPIO)-labelled macrophages [99, 100]. CT can provide information on inflammation by quantifying perivascular fat density (PFD) as has already been shown for the coronary arteries [101]. Baradaran et al [102] found that symptomatic patients had higher mean peri-carotid fat density compared with asymptomatic patients (−66.2 ± 19.2 HU versus −77.1 ± 20.4 HU, *p* value = 0.009). Notably, when comparing non-stenotic ICAs, there was no significant difference between peri-carotid fat density in symptomatic compared with asymptomatic patients (−81.0 ± 13.3 HU versus −85.3 ± 18.0 HU: *p* value = 0.198). The authors suggested that inflammation associated with vulnerable carotid plaques extends beyond the vessel lumen. Another study found that contrast plaque enhancement and PFD are related and that the correlation is stronger for symptomatic patients compared to asymptomatic patients [103].

Nevertheless, despite the mentioned promising advances, these PET-, MR-, and CT techniques are currently limited to research settings rather than clinical practice.

#### Arterial remodeling

**Fig. 5:**
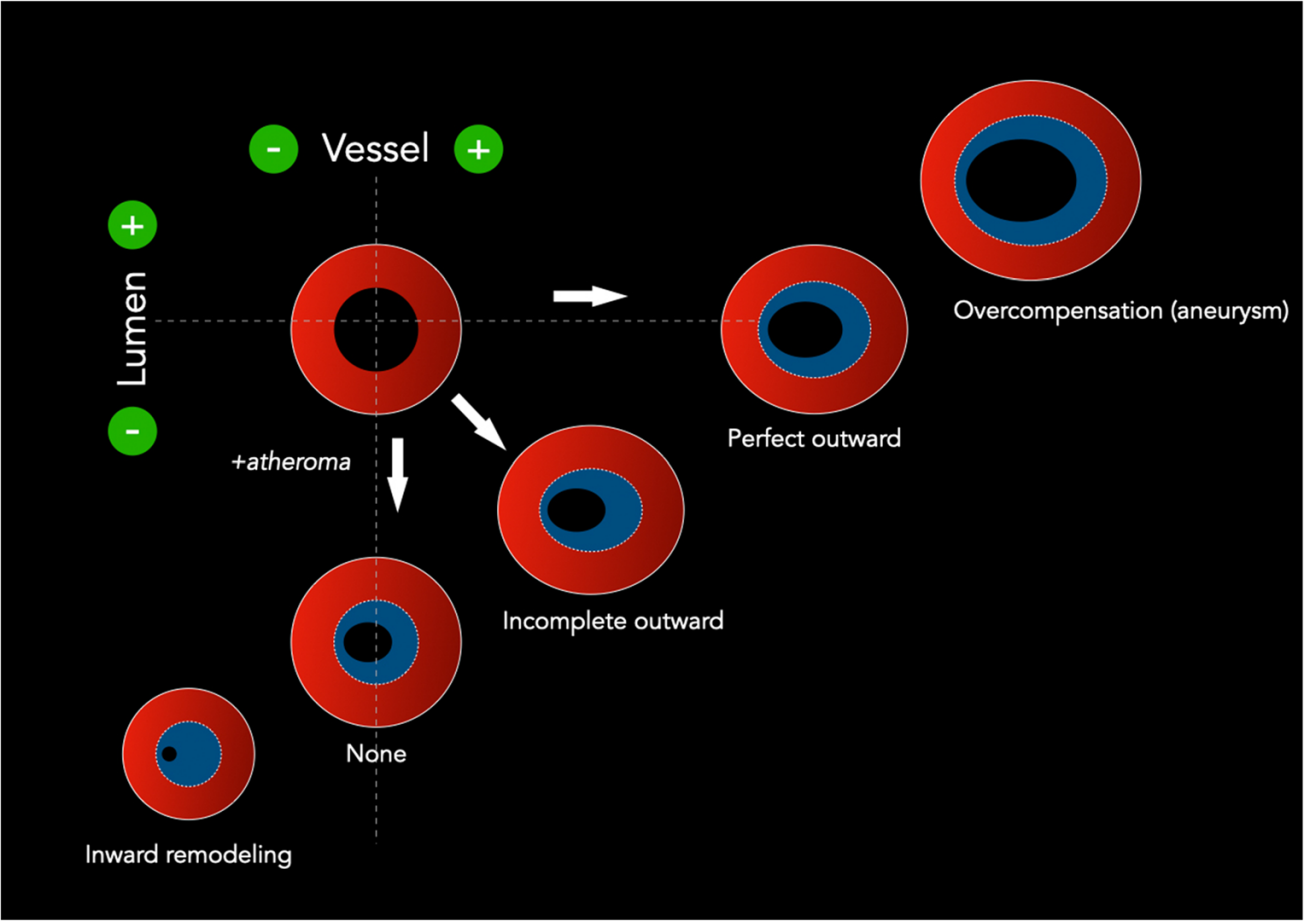
Different forms of vascular remodeling are shown. The relation between the presence of atherosclerotic plaque and its effect on the vessel lumen may significantly vary depending on the accompanying remodeling. As such, even in the presence of significant plaque formation, the repercussion on luminal diameter may be less severe. Illustration modified from reference 105

Spatial variation in atherosclerotic plaque can be observed on imaging. The term “arterial remodeling” was first introduced by Glagov [104] for the coronary arteries. Arterial remodeling refers to a change in vessel size (cross-sectional area) in reaction to atherosclerotic changes. It is possible to distinguish among different types of arterial remodeling [105] (Fig. 5 and Table 4): (*a*) “inward” (negative) remodeling that denotes a reduction in vessel size; (*b*) “outward” (positive) remodeling that denotes an increase in vessel size; (*c*) “concentric” remodeling that denotes a harmonic distribution of the plaque over 360°; and (*d*) “eccentric” remodeling that denotes an abnormal distribution of the plaque in one or more quadrants of the plaque.

**Table 4:** Often-used synonyms for change in vessel size, from reference 105

Carotid plaque remodeling - terminology	
Increase in size	Decrease in size
Outward remodeling	Inward remodeling
Compensatory enlargement	(Paradoxical) shrinkage
Positive remodeling	Negative remodeling
Expansive remodeling	Constrictive remodeling
Glagovian remodeling	Antiglagovian remodeling

**Fig. 6:**
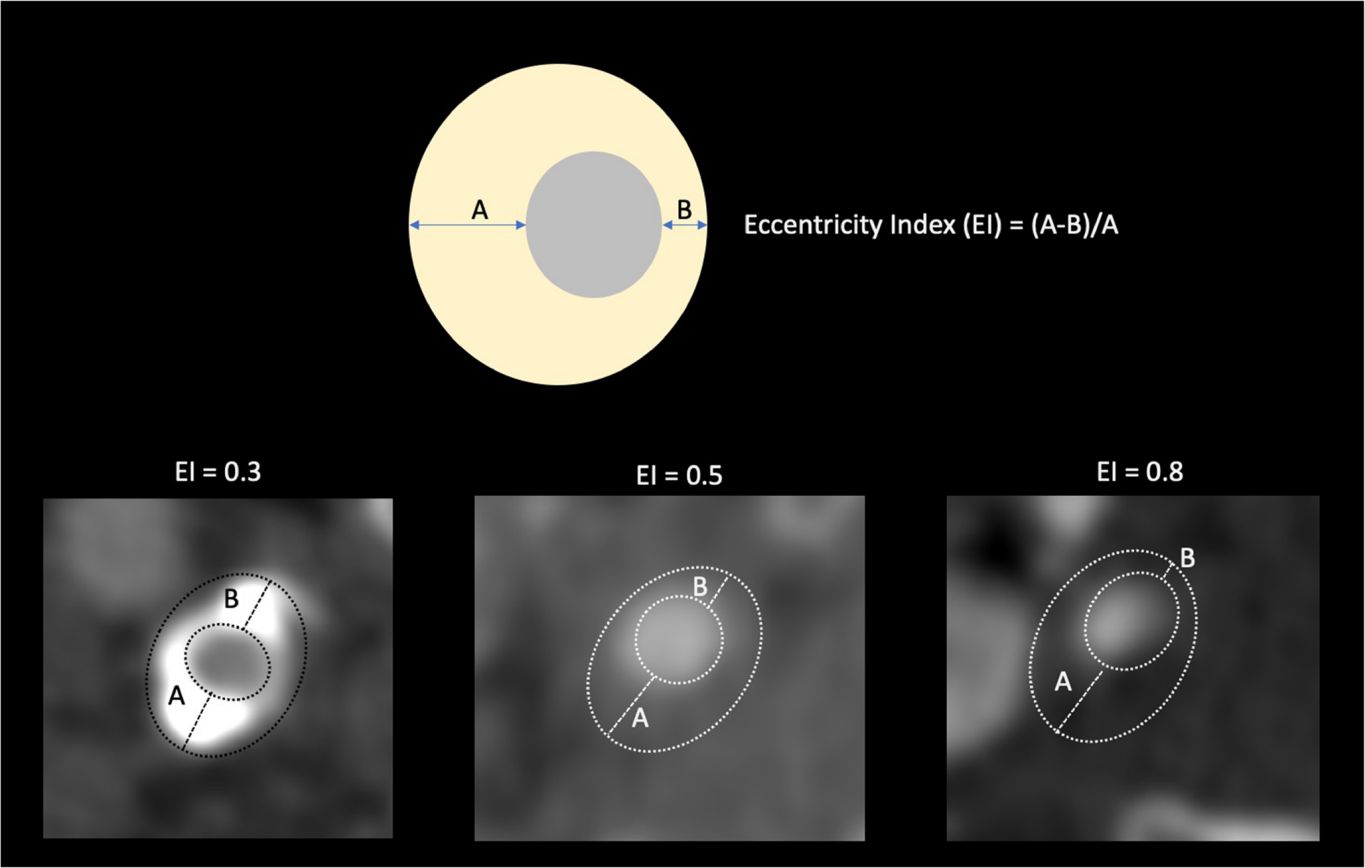
Eccentricity index (EI), reflecting the grade of eccentric positioning of the lumen within the cross-sectional area of the vessel. In a patient with 70% area carotid stenosis, eccentric plaque is reportedly associated with a significantly increased incidence of ipsilateral cerebrovascular events compared with patients with concentric stenosis

When the outward remodeling is present but insufficient to prevent luminal stenosis, it is referred to as “inadequate outward remodeling.” The authors studied the remodeling in the carotid arteries of 108 patients [106] and found that remodeling (measured with the plaque remodeling ratio) was significantly higher in symptomatic (1.64 ± 0.44) compared to asymptomatic patients (1.41 ± 0.5, *p* < 0.05). Similarly, another group [107] investigated 512 internal carotid arteries in 441 patients (introducing the eccentricity index) and found that eccentric plaque was associated with a significantly increased ipsilateral cerebrovascular event rate compared with patients with concentric stenosis (Fig. 6). The eccentricity increases the biomechanical stress to the plaque by determining an increased vulnerability [108] and it was found that it is associated with an increased prevalence of IPH [109]. Initially, positive remodeling was believed to be a protective phenomenon [110], but newer pieces of evidence have demonstrated that it should be considered a parameter of vulnerability [111].

### Vessel- and luminal surface morphology

**Fig. 7:**
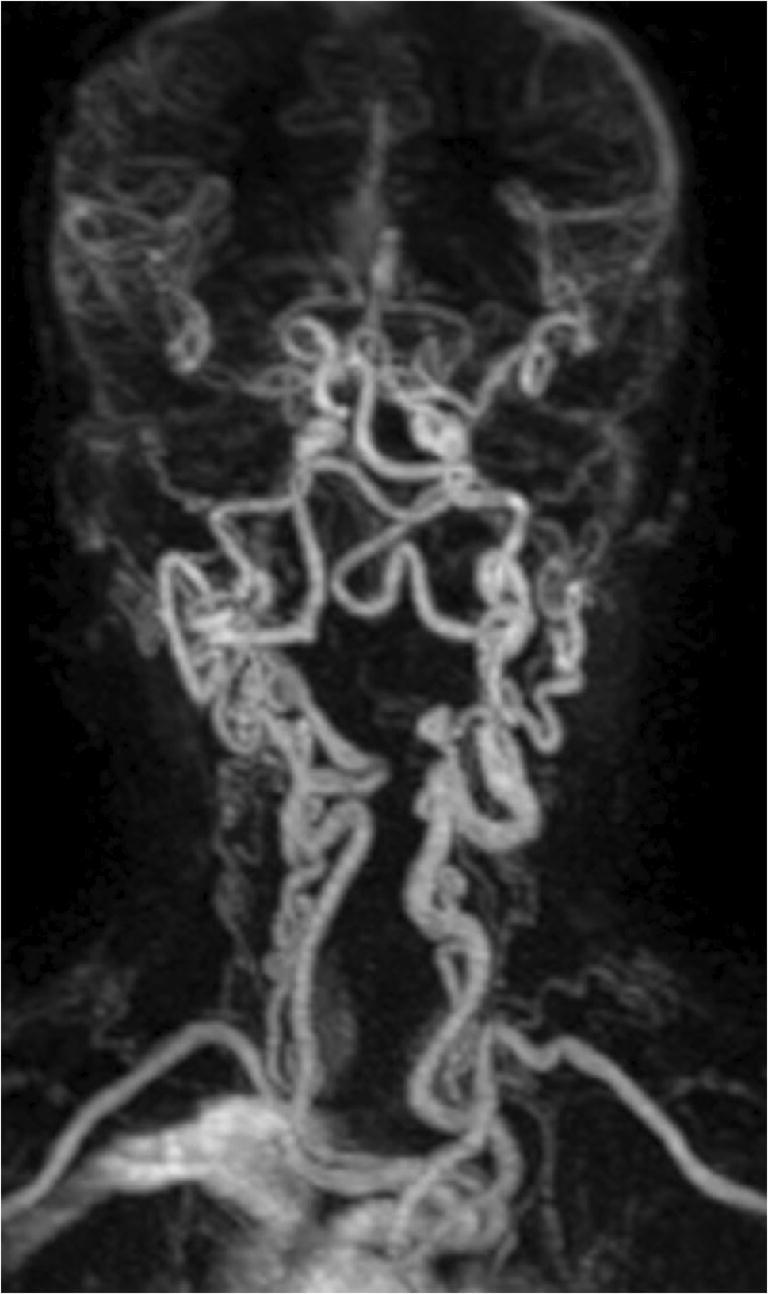
MR angiography examination showing severe kinking and coiling of the common and internal carotid arteries as well as the vertebral arteries in a child with Loeys-Dietz syndrome

*Vessel morphology* can be classified according to the modified criteria of Weibel-Fields and Metz [112–114], which describe the course as tortuous (elongated), kinked (mild, moderate, severe), or coiled when applicable (Table 5). This abnormal morphology can be found in all segments of the common carotid artery (CCA) and ICA (Fig. 7) [115, 116]. The vessel course should be reported since altered vessel tortuosity, in particular, kinking (but not coiling), has shown to be possibly involved in the occurrence of ischemic stroke [116].

**Table 5:** Tortuosity classification, modified from reference 112

Modified criteria of Weibel-Fields and Metz	
Tortuosity	*S- or C- shaped elongation or undulation*
Mild kinking	*Acute angulation with an angle between the two segments forming the kink measured ≥ 60 °*
Moderate kinking	*Acute angulation with an angle between the two segments forming the kink measured 30–60°*
Severe kinking	*Acute angulation with an angle between the two segments forming the kink measured < 30 °*
Coiling	*Elongation or redundancy resulting in an exaggerated S-shaped curve or a circular configuration.*

**Fig. 8:**
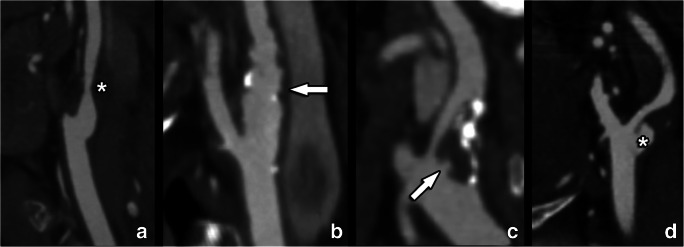
Several possible plaque surface morphologies are illustrated on contrast-enhanced CT examinations. Panel **a** reveals a non-calcified plaque (asterisk) in the internal carotid artery with a completely smooth surface. The second example shows scattered small calcified and non-calcified plaques leading to discrete wall irregularities. A large mixed plaque is shown in panel **c**, leading to not only severe luminal narrowing but also additional proximal ulceration (arrow). Finally, panel **d** shows a large non-calcified plaque with a large ulceration (asterisk) and only moderate luminal narrowing

*Luminal surface morphology* concerns the plaque surface, where the presence of contour irregularities has been shown to significantly contribute to the development of ischemic neurological symptoms due to plaque fragmentation and microthrombi formation (Fig. 8) [117]. Similarly, both NASCET and ECST trials demonstrated the association between plaque luminal surface irregularities and future stroke [118, 119]. However, plaque surface evaluation was previously not included in risk evaluation and consecutive treatment decisions.

**Fig. 9:**
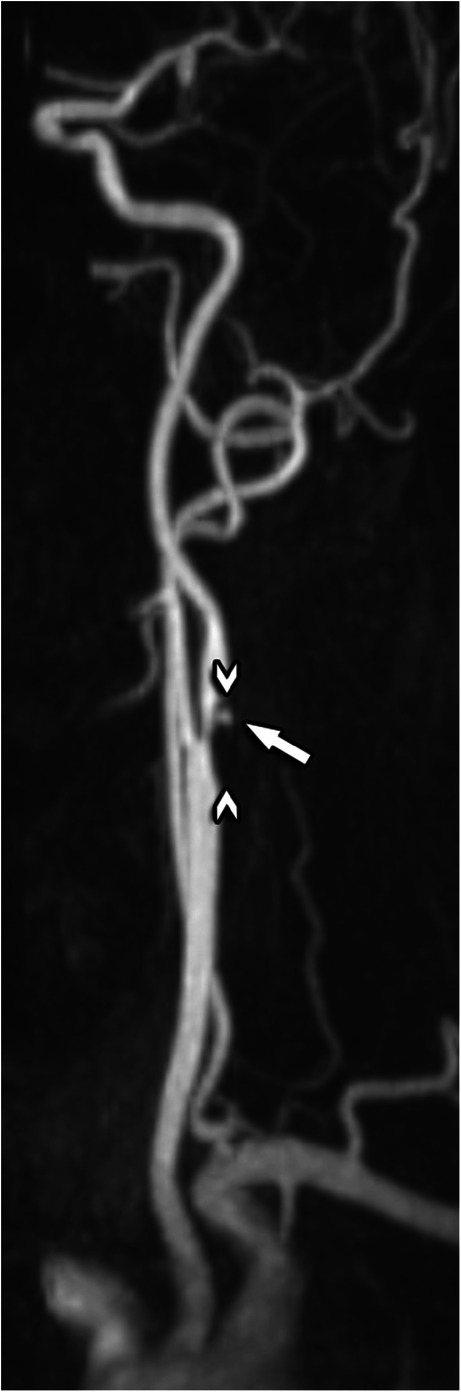
Contrast-enhanced MRA in a 74-year-old man with suspicion of carotid stenosis. An eccentric plaque (arrowheads) can be seen on the transition from the common to the internal carotid artery, with a severe ostial luminal narrowing. The smooth surface of this plaque is interrupted by a small ulceration (arrow)

*Ulceration* is the most important parameter among the possible luminal surface irregularities, given its strong association with cerebrovascular events [120]. However, the exact association between the presence of ulceration and future occurrence of cerebrovascular events is debated, since ulceration could also be considered as a marker of previous plaque rupture rather than the cause of an acute ipsilateral stroke/transient ischemic attack (TIA) [121, 122]. Ulcerations can be evaluated using both CT and MR with varying levels of diagnostic accuracy depending on the used technique [123]. Using CT, the sensitivity is 94% [123], whereas with MR, there is a significant variation depending on the used sequences. In particular, contrast-enhanced MR (CE-MR) detects over one-third more ulcers than non-contrast MR (moving from 55 to 93% sensitivity) **(**Fig. 9) [124].

## Section III: image acquisition

### ESCR Consensus Statement IV

*Both MR and CT are equally suitable for carotid imaging, with both their own strengths and weaknesses. In practice, the choice of imaging modality will depend on local expertise and preference, the required information, the pre-test probability of significant disease, potential contraindications for a specific modality and the availability of imaging equipment. In general, CT provides a vast amount of information on luminal stenosis and the underlying plaque in one short examination, is readily available and does not require advanced technical skills. Therefore, it can be recommended as the initial choice in a symptomatic patient being evaluated for carotid artery disease.*

In this section, we provide consensus guidelines for an optimized approach to the carotid arteries by means of CT and MR, keeping in mind that protocols will vary according to the CT and MR scanner technology available. Therefore, consensus proposals are made considering application across a broad spectrum of scanner manufacturers and do not require specialized software or research applications for clinical implementation [125].

A modern carotid imaging study should be able to provide information on the following topics:

1. Comprehensive anatomical and morphological assessment of the vessels
2. Presence and quantification of the degree of stenosis
3. Assessment of luminal surface morphology
4. Characterization of the plaque composition (variable information according to the use of the CT or MR)
5. Other parameters, despite not being mandatory according to the current level of evidence, should be incorporated when possible.

### CT

A basic guideline for a CT-scan protocol is given in Table 6.

**Table 6:** CT-scan protocol. Suggested scan parameters and general guidelines are given

CT scan protocol—parameters		
Feature	Value	Additional comments
Coverage	Aortic arch to Circle of Willis	Always evaluate degree of atherosclerosis and morphology of the aortic arch and potential variants of the circle of Willis
Scan mode	Helical (may vary on scanner type)	Protocol must be adjusted according to manufacturer guidelines (singe vs dual-source systems)
Scan direction	Caudo-cranial	Not commonly used, small anatomic coverage, to be considered when intravenous contrast is contraindicated
Start of acquisition	Bolus tracking on the aortic arch	Local experiences may vary
Collimation	0.6 × 64 (or better)	Depends on the scanner detector configuration
Pitch	Depending on the scanner type, typically < 1	
kV	120	100 kV is not recommended for examinations, as kV influences plaque attenuation values.
mA	350	Anatomy-based modulation may be used
FOV	200 mm, centered on cervical arteries	A well-centered FOV may improve resolution and lower dose
Filter	Medium to sharp	Local experience may vary; Visual effect of filter may be influenced by chosen iterative reconstruction techniques and other noise-reductions algorithms
Slice thickness	< = 1 mm for secondary raw data set, 3 mm for reading data set	Secondary raw data set is used for detail reviewing and post-processing
Reconstruction interval	50 % of chosen slice thickness for the secondary raw data set	No overlap is necessary for the reading of 3 mm datasets
CT-scan protocol—general guidelines		
Patient preparation	Check for usual CT-related contra-indications	
IV access	Antecubital vein (right arm preferred)	
Gadolinium-contrast concentration	> = 300 mg iodine/mL	
Contrast volume	30–50 mL depending on body weight	
Injection rate / saline flush	> = 4 mL/s; 50 cc saline flush	
Evaluation of neovascularization	A non-enhanced scan is necessary, identical scan parameters	
Post-processing	Review of axial unprocessed images	
	Curved-MPR images along a complete trajectory	
	VR-images for complex/tortuous anatomy	
Reporting	Structured reporting recommended	

#### Technical parameters

An isotropic voxel size of ≤ 0.6 mm is mandatory [126, 127]. This is not only necessary for accurate visualization of the lumen and potential stenosis, but also for high-resolution plaque evaluation and its different components. All modern CT equipment can meet this requirement without difficulty. Tube current mA settings are usually modulated according to the anatomy, with a general value between 300 and 350 mA commonly applied for most patients [128]. Caution must be taken with some CT scan models, as higher values may result in the scanner using a larger focus, resulting in less spatial resolution for the chosen field of view.

Tube voltage is a key parameter that influences attenuation values. As such, using different kV values will result in different attenuation values of the same tissue. Therefore, the same tube voltage should be used or settings that guarantee optimal reproducibility [128]. The 120-kV tube voltage setting is widely used in the literature with the best results in terms of SNR for consistent tissue assessment compared with other settings. The use of lower kV values would, on one hand, improve the attenuation of the opacified lumen but conversely slightly reduce the visualization of periluminal tissues on the other hand. Nevertheless, when no plaque component measurements are required (e.g., follow-up examinations), and depending on the clinical context, lower kV settings and application of modern noise reduction mechanisms (e.g., iterative reconstruction techniques) can be successfully applied, delivering good diagnostic performance at lower radiation exposure levels [129–131].

For routine evaluation, a standard soft-tissue-centered reconstruction kernel is typically used, the specifics of which vary among manufacturers and CT models. These kernels offer a good starting point delivering a balanced image reproduction of different calcified- and non-calcified tissue components with good SNR levels. On occasion, a sharper kernel can be applied when evaluating heavily calcified plaques, delivering a better delineation of the calcifications against the contrast-enhanced vessel lumen. However, this often comes at the expense of an increase in image noise; therefore, it is not recommended to routinely apply sharper kernels in all patients. Image noise can be countered by iterative reconstruction techniques, which can also affect image characteristics in different ways. Users can combine all these different options into specific programs tailored to their tastes.

It is important to underline that the evaluation of atherosclerotic arterial plaque characteristics is currently based on qualitative biomarkers. However, the reproducibility of such findings is suboptimal as attenuation values are influenced by multiple factors (from the kV to the body mass index of the patients) [132]. Therefore, standardization across different manufacturers with cross-calibration is required to establish a quantitative evaluation of carotid CT. In this scenario, the Quantitative Imaging Biomarker Alliance (QIBA) profile is trying to identify standard imaging parameters and characteristics. A white paper on this area is forthcoming.

#### Anatomic coverage

Coverage is required from the aortic arch to the circle of Willis. It is fundamental to include the entire aortic arch to exclude the presence of atherosclerotic and potentially embolic thrombus (mandatory assessment in stroke patients) and for the assessment of arch anatomy (mandatory for carotid stenting procedures). The patency of the origins of all cervical arteries must also be properly checked.

#### Contrast injection protocol

A CT study of the carotid arteries should be accurately timed to be executed in the arterial phase. This is important, as a good contrast between lumen, surrounding plaque, and soft tissue is essential for an adequate evaluation. The contrast material should be administered, pre-heated at 37° C, with an iodine-delivery rate (IDR) of 1400–2000 mg/s and a recommended concentration of ≥ 300 mg/mL iodine content. A bolus-triggering technique to accurately visualize the contrast arrival at the level of the aortic arch for optimal timing is recommended. The total contrast volume usually varies between 30 and 50 mL, function of the iodine concentration, scanner technology, required density, and—if possible—the left ventricular ejection fraction. The patient should be in a supine position with his/her arms along the body. Additionally, patients should also be instructed not to breathe and not to swallow during the examination. It is beneficial to instruct the patients about this prior to the start. Contrast administration should be performed via an 18–20-gauge intravenous catheter inserted into the antecubital vein.

For assessment of carotid plaque neovascularization, a biphasic approach (unenhanced scan followed by CT angiography) is required. In this case, energy levels (tube voltage (kV) and tube current (mAs)) should be identical to be able to accurately compare differences in attenuation values. However, this approach leads to increased radiation exposure for the patient. This can be partially countered by limiting the range of the unenhanced scan to the expected carotid bifurcation level. Nevertheless, identification of the correct scan area remains challenging, especially in patients with anatomical variations (e.g., high carotid bifurcation).

The use of dual-energy/spectral CT imaging may provide additional information to conventional CT [133], delivering virtual iodine maps and virtual unenhanced scans from a single contrast-enhanced acquisition [134], as such alleviating the need for extra radiation exposure. However, dual-energy / spectral CT techniques have other challenges which are beyond the scope of this paper.

#### Post-processing

**Fig. 10:**
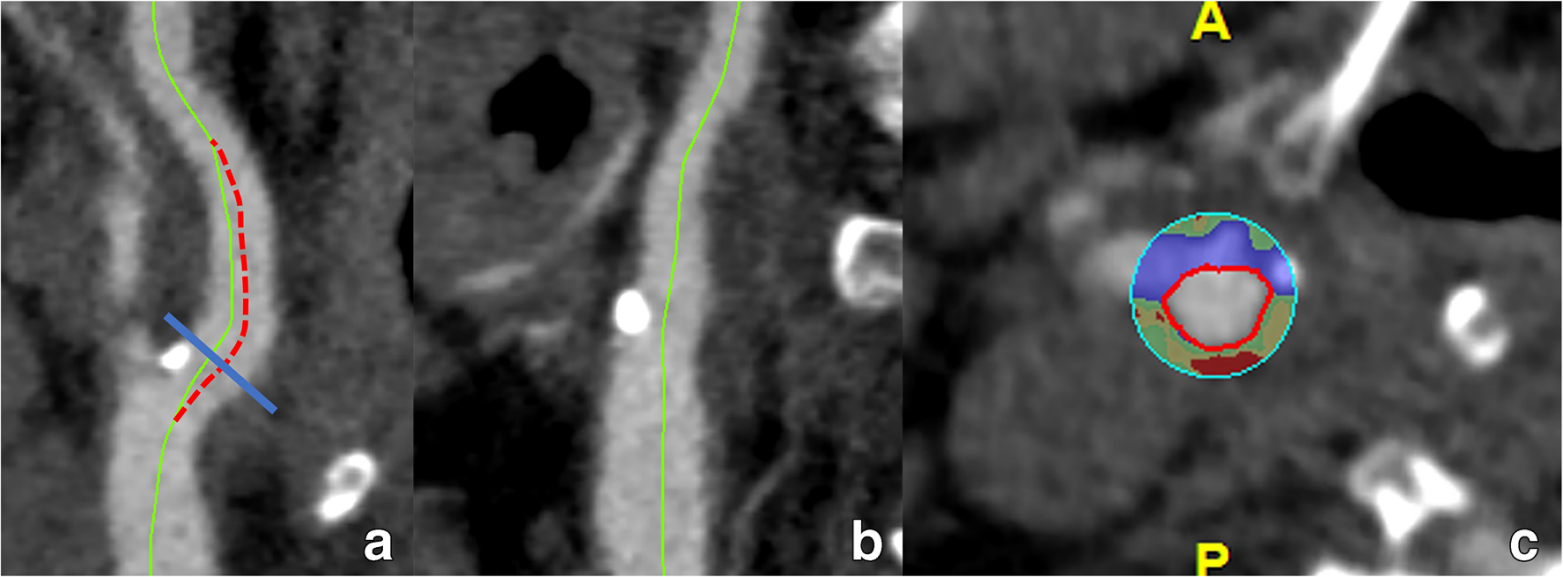
The c-MPR-technique and the importance of an adequately positioned centerline. A c-MPR image of the carotid bulb is shown (**a**, **b**). The original centerline (green) must be checked in all angles. While it nicely follows the center of the lumen in a frontal view (**b**), it deviates from it in a sagittal view (**a**), most notably on the transition from the common carotid artery to the internal carotid artery and more distally in the carotid bulb. As such, it produces in the resulting cross-sectional image an eccentrically positioned lumen (**c**), which can lead to wrong conclusions. Manual correction (red dotted line in a) is necessary to avoid interpretation mistakes

**Fig. 11:**
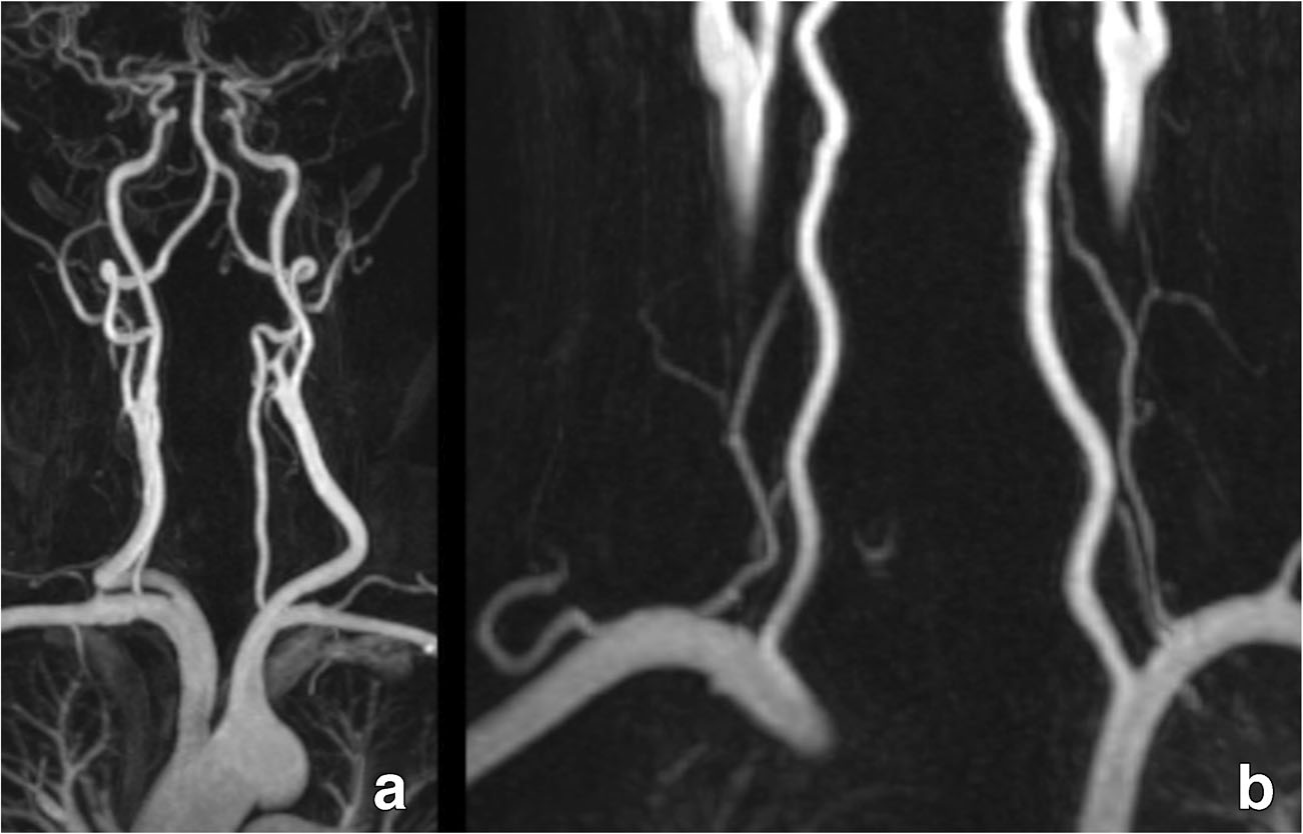
Contrast-enhanced gradient echo T1-weighted MR angiography of the cervical arteries. Conversely to CT angiography, MIP-images in MR angiography provide direct visualization of the whole trajectory of the carotid- and cervical arteries without bone- or other soft tissue superposition (**a**). This allows excellent visualization of segments which are near bones at the base of the neck, such as the origin of the vertebral arteries (**b**). MIP, maximum intensity projection

Multiplanar review of all unprocessed native image sets remains the starting point for each evaluation. For further processing, we recommend a post-processing workflow set including maximum intensity projections (MIP) and curved multiplanar reformations (CMPR). CMPR are to be used for the selection of the point of maximum stenosis and for the identification of the denominator for the NASCET/ECST assessment. However, the correct position of the centerline in the lumen must always be checked, as an asymmetric location outside the center of the vessel will lead to wrongly reformatted images and consequently potential evaluation errors (Fig. 10). Therefore, caution is always required when interpreting CMPR-images, especially in complex anatomical situations with tortuous branches. MIP images are used for anatomy assessment, especially useful in MR (Fig. 11). In some cases, in order to better illustrate the anatomical relationship between arteries and other tissues, the use of volume rendering (VR) images could be considered (e.g., Eagle syndrome). The stenosis degree should be quantified in CMPR views.

### MR

A basic guideline for a MR scan protocol is given in Table 7.

**Table 7:** MR-scan protocol. Suggested scan parameters and general guidelines

MR scan protocol—parameters					
Anatomical target	Sequence*	Scan range	IV contrast	Signal characteristics	Additional comments
Lumen / ulceration visualization	3D GRE T1WI	Aortic arch to Circle of Willis	Yes	T1-shortening of contrast agents enhances arteries against darker background	Most common method for CE-MRA. Commonly pre- and post-contrast acquisitions are performed
	Time-resolved 3D GRE T1WI	Aortic arch to Circle of Willis	Yes	Same principle, but multiple short acquisitions provide dynamic flow information	May be used when patient cooperation is limited, incorporates non-contrast acquisition within same acquisition
	3D TOF	Carotid bifurcation	No	High signal of moving voxels in selected volume	Not commonly used, small anatomic coverage, to be considered when intravenous contrast is contraindicated
Intraplaque hemorrhage (IPH)	IR-TFE/SPGR (MP-RAGE)	Carotid bifurcation	No	High signal of blood degradation products within plaque	Most important detectable vulnerable plaque component. Presence is associated with increased future risk for ipsilateral stroke
Lipid-rich necrotic core (LRNC)	TSE/FSE T2WI	Carotid bifurcation	No	Hypointense on T2WI	May be used with intravenous contrast is contra-indicated
	Non-enhanced and CE-T1WI	Carotid bifurcation	Yes	Hyperintense on T1WI	LRNC does not enhance on CE-T1WI sequences
Fibrous cap (FC)	3D TOF	Carotid bifurcation	No	Hypointense with varying thickness	An intact FC has a smooth and regular surface
	CE-T1WI	Carotid bifurcation	Yes	Intact FC: enhancing and smooth band against dark lumen; thinned FC: smooth but not-enhancing band; ruptured FC: irregular surface non-enhancing band	Intact FC: enhancing and smooth band against dark lumen; thinned FC: smooth but not-enhancing band; ruptured FC: irregular surface non-enhancing band
MR scan protocol—general guidelines					
Field strength		At least 1.5T, preferably 3T systems			
Coils		If available, dedicated carotid coils must be used			
Patient preparation		Check for usual MR-related contra-indications			
IV access		Antecubital vein (right arm preferred)			
Gadolinium-contrast concentration		> = 0.5 mol/L Gadolinium concentration. High relaxivity agents are preferred.			
Contrast volume		6–15 mL depending on body weight			
Injection rate / saline flush		2 mL/s; 50 cc saline flush			
2D vs 3D sequences		3D sequences are recommended, but local expertise may vary			
CE-MRA Post-processing		Review of raw non-subtracted contrast-enhanced images MIP images processed on work station VR-images for complex/tortuous anatomy			
Reporting		Structured reporting recommended			

*GRE, gradient echo; *TOF*, time of flight; *IR-TFE*, inversion recovery turbo field echo; *SPGR*, spoiled gradient recalled; *TSE*, turbo spin echo; *SE*, spin echo; *CE*, contrast-enhanced

#### Technical parameters

As with CT, the fundamental requirements of a MR protocol include the following: (*a*) isotropic voxel size ≤ 1 mm with ideally 0.5 mm in-plane resolution; (*b*) optimal blood suppression in plaque burden visualization sequences; and (*c*) adequate SNR in all sequences for reliable evaluation. The MR protocol may consider either 2D or 3D or a combination of sequences that meet the minimum requirements set forth above. 3D sequences are preferred as they can detect plaques extending beyond the 4-cm coverage centered on the bifurcation. Although it is known that adequate image quality can be obtained with 1.5T [135], the use of high-field strength 3T scanners and dedicated carotid coils is recommended for improved signal-to-noise ratio (SNR).

#### Anatomic coverage

The longitudinal coverage is two-fold: 3–4 cm centered on the carotid bifurcation for plaque analysis (MR) and from the aortic arch to the circle of Willis for the overall assessment of supra-aortic vessels (MRA).

#### Contrast injection protocol

Examinations can be performed with or without the administration of contrast material. In the absence of contraindications for Gadolinium-based contrast agents, contrast-enhanced MR is recommended for the detection and quantification of LRNC, the delineation of the fibrous cap, and neovascularization assessment. Contrast material also increases MR sensitivity for the detection of ulcerations. Finally, contrast-enhanced MR angiograms provide an overview of the anatomical situation and DSA-like images.

Similar to CT, contrast injection is preferably performed through an antecubital vein. However, contrast injection rates can be lower than their CT counterparts, with excellent results achievable with a 1–2 mL/s injection speed adapted to body habitus. The use of higher Gadolinium concentrations is recommended (1.0 mmol/mL as they improve SNR for the same contrast volume compared with agents with lower concentrations or achieve the same SNR at half the volume.

MR protocols suitable for 2D/3D approaches, with or without intravenous contrast enhancement, can be found in Table 7.

#### Plaque composition

2D multi-contrast MR protocols consisting of T1-, T2-, PD-weighted black-blood imaging, and time-of-flight angiography (TOF) are the standard clinical tool for plaque characterization [136]. However, accurate measurement of plaque morphology requires high isotropic resolution in all three spatial directions. In the current 2D MR protocols, the slice resolution is limited compared with in-plane resolution (2 to 3 mm vs. 0.6 to 0.7 mm). For this reason, the use of 3D MR protocols is recommended.

MR imaging of the carotid arteries should offer information on both the lumen morphology of the vessel and the morphology of the underlying plaque. MR contrast between different tissues is mainly caused by their different proton density (PD) or relaxation time constants [137] and thus allows to distinguish between different tissues within the plaque according to their signal intensities (Table 3). A study demonstrated that the highest correlation for IPH area with histologic findings was obtained with magnetization-prepared rapid gradient-echo imaging (rho value = 0.813), followed by time-of-flight (rho value = 0.745) and fast spin-echo (rho value = 0.497) imaging [138]. Three-dimensional magnetization-prepared rapid gradient echo has recently been recommended as the sequence of choice for detecting IPH [125] and three-dimensional magnetization-prepared rapid gradient-echo imaging might improve the detection of small IPHs [139]. Other plaque features associated with an increased risk that should be identified in a MR exam are the presence of a large LRNC, a thin or ruptured fibrous cap and the plaque burden or plaque volume (Table 8).

**Table 8:** Overview of different plaque components, their clinical significance, and preferred imaging modality

Characteristics of different plaque components				
Plaque feature	Pathophysiological impact	Feature of plaque vulnerability	Preferred imaging modality	Reported in clinical practice
Fibrous cap	Thinning of the fibrous cap increases the risk of plaque rupture	++	MR	No
Calcifications	While overall considered a sign of plaque stability, conversely the type and chemical composition of calcium in atheromatous plaques can potentially increase plaque vulnerability	- (but debated)	CT	Yes
Intraplaque hemorrhage	Most important imaging biomarker for plaque instability. It is independent of stenosis severity, associated with acute events, and also with an increased risk for ipsilateral future ischemic events in both symptomatic and asymptomatic subjects.	+++	MR	Yes
Lipid-rich necrotic core	An increased amount of intraplaque lipid is associated with elevated cerebrovascular risk.	++	MR	Yes
Maximum wall thickness	Maximum plaque thickness (measured in mm) is a predictor of cerebral ischemic events	+	CT/MR	Research setting
Plaque volume	Larger volume is associated with increased vulnerability and occurrence of future cerebrovascular events	+	CT > MR	Research setting
Plaque neovascularization	Denser vasa vasorum network is associated with symptomatic disease	+	CT/MR	Research setting
Plaque- and perivascular fat inflammation	Inflammation is associated with cerebrovascular events	+++	[^18^F]FDG PET-CT / MR	Research setting
Plaque remodeling	Remodeling refers to cross-sectional vessel area changes in reaction to atherosclerotic changes. Inadequate outward (positive) eccentric remodeling is associated with symptomatic disease and increased ipsilateral cerebrovascular events.	++	CT/MR	Yes

#### Blood suppression

Blood suppression is a key feature for optimal image quality in MR of the carotid arteries, as adequate suppression of the bright signal from blood in the vessel lumen is required to prevent ghosting of blood signal into the artery wall and partial volume contamination [137]. It has been reported that the use of local transmission coils limits the efficient suppression of inflowing blood. To counter this, it has been suggested to extend the coverage of the radiofrequency transmission coils to ensure efficient black-blood imaging with good wall/lumen definition.

Blood flow suppression and motion artifacts secondary to long scan times are challenging for 3D black-blood carotid MR implementations. Traditional black-blood preparations such as inflow suppression and double inversion recovery developed for 2D MRI do not provide adequate flow suppression for 3D MRI. To ensure effective blood suppression to accurately identify plaque lumen boundaries, MSDE/FSD flow suppression [140] is required for 3D SPACE/CUBE/VISTA and after the injection of contrast material, the use of MSDE or DIR/QIR flow suppression is suggested [141, 142].

Based on current evidence and guidelines, we recommend TI of 250 ms for 3-T scanners for a TR triggered at 1 RR interval, with a good flow suppression beginning 5 min after injection [125].

### Advances in artificial intelligence

**Fig. 12:**
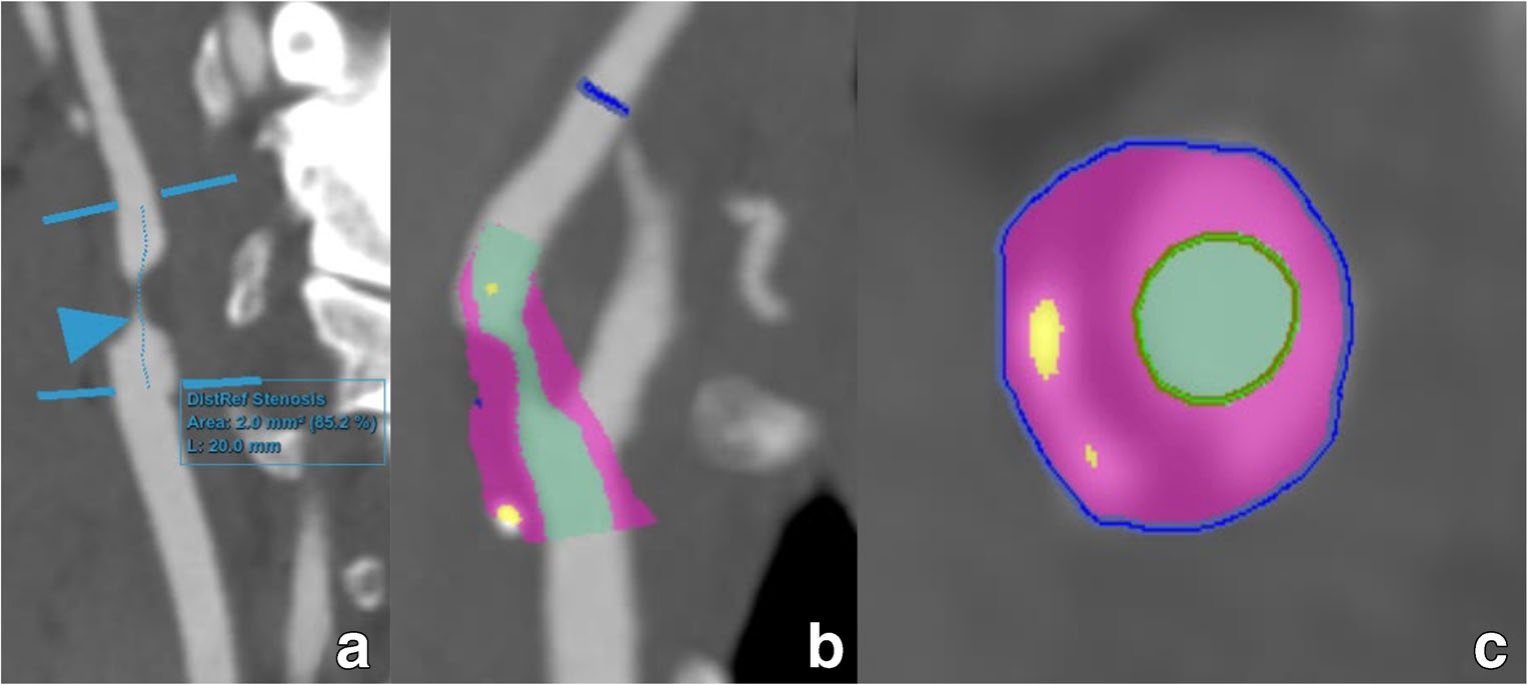
Contemporary post-processing examples of stenosis and plaque analysis software applications. A severe internal carotid artery stenosis is shown on a c-MPR image (**a**) obtained through automated tracking software along the trajectory of this vessel. Subsequent analysis using AI-driven software tools reveals an area stenosis of 85%. Area stenosis measurements on CT images are difficult to implement in routine practice due to often necessary manual corrections, increasing post-processing time. AI tools may, as in this example, improve analysis time while delivering more accurate results. Further analysis using color-coding of different plaque components in two views (**b**, **c**) reveals that while the plaque is mainly composed of non-calcified components (purple), there are some scattered plaque calcifications (yellow). Note also the very good delineation of the lumen (green) vs the surrounding plaque (purple), a result obtained through an AI algorithm delivering more accurate results than previously possible

Artificial intelligence, and machine learning (ML) in particular, has a growing impact on the field of biomedical imaging [143] (Fig. 12). The contributions of ML in the context of carotid artery disease can be assigned to four broader categories. First, *carotid artery segmentation* [144–147], which is the basis for many secondary analyses, provides potential for more comprehensive analyses of vessel anatomy and pathology. For example, Tsakanikas et al proposed a U-net model to produce a 3D meshed model of the carotid bifurcation and branches using multispectral MR image series and reported an accuracy of 99.1% for lumen area [144]. The second is the *carotid plaque detection and segmentation* [148, 149]. Biswas et al applied a two-stage deep learning (DL) model to detect plaques and among others measure total plaque area [148]. In general, ML algorithms of this category can help radiologists to reduce the number of missed findings and to provide more detailed evaluations to referring physicians (e.g., area and volume measurements instead of diameters). The third is *carotid plaque characterization* [150–152]. Skandha et al developed multiple DL/ML approaches to classify symptomatic and asymptomatic carotid plaques [151]. The fourth is *prediction of plaque rupture or patient risk* [148, 150, 153]. Araki et al used texture features derived from carotid walls as input to a ML model to stratify patients according to their risk for a stroke [154]. These models can utilize the full information contained in imaging data.

Integration of AI solutions into clinical workflows remains however challenging. Currently, quantitative plaque analysis CT software, while available, is not yet used in clinical practice as they prove to be often time-consuming, impacted by e.g. reconstruction parameters. Also, they are not clinically validated, often lacking robust evidence that they could offer a significant impact in terms of risk stratification. For the moment, the development of AI applications in the field of biomedical imaging is highly dynamic. Over time, AI tools will potentially improve in accuracy and reduce analysis time, leading the way for the more routine use of quantitative plaque analysis in future clinical practice.

### Choice of modality: CT or MR?

Both CT and MR can provide a complete overview of the full vasculature from the aortic arch to the circle of Willis, a distinct advantage compared to duplex ultrasound. Sensitivity and specificity for the detection of high-grade carotid stenosis are high for both modalities. The main advantages of MR are the lack of blooming artifacts caused by extensive calcifications, the ability to analyze vessel wall components and characterize plaques in detail, as well as the lack of radiation exposure. Disadvantages of MR include the use of gadolinium contrast agents, the examination duration, and contraindications to MR in certain patients.

The main advantages of CT are defined by the high spatial resolution, direct visualization of both luminal stenosis and carotid plaque with calcifications, fast acquisition, and broad availability including the widely accepted use f CTA of the head and neck vessels in the acute stroke work-up. Furthermore, CT has shown an exciting innovation, and next-generation scanners might provide even more in-depth information about plaque components. Disadvantages of CT include radiation exposure and the need for iodine contrast media which limits usability in patients with severely impaired renal function.

Previous interventions in neighboring anatomy like orthodontic prosthetics can on occasion hamper visualization of the carotid arteries. The extend of the generated artifacts varies with the location, type, and amount of the material used. Some manufacturers have developed proprietary solutions to reduce these artifacts on CT, while in general other more widely available novel reconstruction methods like iterative reconstruction can also help in reducing image quality degradation [155]. On MRI, the generated artifacts vary with the chosen sequences. As a general rule, GE-sequences intravoxel dephasing artifacts can be reduced by decreasing voxel size and shortening echo time. A well-chosen and aligned field-of-view can also help in decreasing artifacts from previous dental work.

## Discussion and conclusion

An optimal imaging strategy for the characterization of the pathology of the carotid arteries, with special emphasis on atherosclerotic disease, is crucial given the different therapeutical approaches available (drug therapy, carotid endarterectomy, carotid artery stenting). In the past, most therapeutical decisions were based on the degree of stenosis and the presence of plaque surface irregularities. Today, thanks to the improved knowledge of the pathology and biology of atherosclerosis as well as improvements in imaging techniques, more information can be obtained and should consequently be offered in the routine clinical report.

Therefore, this writing group has explored the scenario and emerging role of CT and MR imaging as the most used advanced imaging modalities approach [20]. We are aware that other imaging techniques such as duplex ultrasound and nuclear medicine also play a role in the imaging of carotid arteries. It was decided not to include ultrasound because of its already well-defined role as a first-line approach with extensive and dedicated literature covering technical aspects and reporting. Nuclear medicine techniques were not included in detail because of their current, limited, application in clinical practice.

This consensus statement aimed to provide an in-depth overview of established and emerging imaging-derived biomarkers, to better describe carotid artery diseases as well as to predict the individual risk of ischemic stroke. Despite the existing evidence about most of these parameters, the current clinical treatment decision guidelines do not take advantage of this knowledge, whereas it is the belief of this writing group that a comprehensive summary of existing parameters is needed. This statement presents the actual standard of practice in carotid artery imaging and provides guidelines for both standardized image acquisitions and for standardized reporting. Given the fast-evolving technique, especially in modern CT techniques, an update will be published within the next 5 years.

## Abbreviations

^18^F: 18-fluorodeoxyglucose
AHA: American Heart Association
CCA: Common carotid artery
CMPR: Curved multi planar reformations
CT: Computed tomography
CTA: Computed tomography angiography
DIR/QIR: Double inversion-recovery / quadruple inversion-recovery
EI: Eccentricity index
FC: Fibrous cap
FSD: Flow-sensitive dephasing
ICA: Internal carotid artery
IDR: Iodine delivery rate
IPH: Intraplaque hemorrhage
LPNC: Lipid-rich necrotic core
MIP: Maximum intensity projection
MR: Magnetic resonance
MRA: Magnetic resonance angiography
MSDE: Motion-sensitized driven-equilibrium
PD: Proton density
PET: Positron emission tomography
PFD: Perivascular fat density
SNR: Signal-to-noise ratio
SPACE: Sampling perfection with application optimized contrasts using different flip angle evolutions
T1W: T1-weighted
TOF: Time of flight
US: Ultrasound
USPIO: Ultrasmall superparamagnetic iron oxide
VISTA: Volume isotropic turbo spin-echo acquisition
VR: Volume rendering

## Declarations

### Guarantor

The scientific guarantor of this publication is Luca Saba, Department of Radiology, University of Cagliari, Cagliari, Italy.

### Statistics and biometry

No complex statistical methods were necessary for this paper.

### Methodology

• scientific society consensus statement
