# Correction to: State-of-the-art CT and MR imaging and assessment of atherosclerotic carotid artery disease: standardization of scanning protocols and measurements—a consensus document by the European Society of Cardiovascular Radiology (ESCR)

**DOI:** 10.1007/s00330-022-09246-9

**Published:** 2022-11-28

**Authors:** Luca Saba, Christian Loewe, Thomas Weikert, Michelle C. Williams, Nicola Galea, Ricardo P. J. Budde, Rozemarijn Vliegenthart, Birgitta K. Velthuis, Marco Francone, Jens Bremerich, Luigi Natale, Konstantin Nikolaou, Jean-Nicolas Dacher, Charles Peebles, Federico Caobelli, Alban Redheuil, Marc Dewey, Karl-Friedrich Kreitner, Rodrigo Salgado

**Affiliations:** 1 Department of Radiology, University of Cagliari, Cagliari, Italy; 2 Division of Cardiovascular and Interventional Radiology, Department of Biomedical Imaging and Image-Guided Therapy, Medical University of Vienna, Vienna, Austria; 3 Department of Radiology, University Hospital Basel, University of Basel, Basel, Switzerland; 4 BHF Centre for Cardiovascular Science, University of Edinburgh, Chancellor’s Building, 49 Little France Crescent, Edinburgh, EH164SB UK; 5 Edinburgh Imaging Facility QMRI, University of Edinburgh, Edinburgh, UK; 6 Policlinico Umberto I, Department of Radiological, Oncological and Pathological Sciences, Sapienza University of Rome, Rome, Italy; 7 Department of Radiology & Nuclear Medicine, Erasmus MC, Rotterdam, The Netherlands; 8 Department of Radiology, University of Groningen, University Medical Center Groningen, Hanzeplein 1, 9713 GZ Groningen, The Netherlands; 9 Department of Radiology, Utrecht University Medical Center, Heidelberglaan 100, 3584 CX Utrecht, The Netherlands; 10 Department of Biomedical Sciences, Humanitas University, Via Rita Levi Montalcini 4, Pieve Emanuele, 20072 Milan, Italy; 11 IRCCS Humanitas Research Hospital, Via Manzoni 56, Rozzano, 20089 Milan, Italy; 12 Department of Radiological Sciences - Institute of Radiology, Catholic University of Rome, “A. Gemelli” University Hospital, Rome, Italy; 13 Department of Diagnostic and Interventional Radiology, University of Tuebingen, Tübingen, Germany; 14 Department of Radiology, Normandie University, UNIROUEN, INSERM U1096 - Rouen University Hospital, F 76000 Rouen, France; 15 Department of Cardiothoracic Radiology, University Hospital Southampton, Southampton, UK; 16 University Clinic of Nuclear Medicine Inselspital Bern, University of Bern, Bern, Switzerland; 17 Institute of Cardiometabolism and Nutrition (ICAN), Paris, France; 18 Department of Cardiovascular and Thoracic, Imaging and Interventional Radiology, Institute of Cardiology, APHP, Pitié-Salpêtrière University Hospital, Paris, France; 19 Laboratoire d’Imagerie Biomédicale, Sorbonne Universités, UPMC Univ Paris 06, INSERM 1146, CNRS, 7371 Paris, France; 20 Department of Radiology, Charité - Universitätsmedizin Berlin, Charitéplatz 1, 10117 Berlin, Germany; 21 Department of Diagnostic and Interventional Radiology, University Medical Center, Mainz; Langenbeckstraße 1, 55131 Mainz, Germany; 22 Department of Radiology, Antwerp University Hospital & Antwerp University, Holy Heart Lier, Belgium

**Correction to: European Radiology**

10.1007/s00330-022-09024-7

The original version of this article, published on 04 October 2022, unfortunately contained a mistake. The first names of all authors were given as initials and have now been written out in full. The original article has been corrected.

